# Reproductive health and the politics of abortion

**DOI:** 10.1186/s12939-020-1157-1

**Published:** 2020-03-17

**Authors:** Astrid Blystad, Haldis Haukanes, Getnet Tadele, Karen Marie Moland

**Affiliations:** 1 Global Health Anthropology Research Group, Centre for International Health, Department of Global Public Health and Primary Care, University of Bergen, Bergen, Norway; 2 Centre for Intervention Science in Maternal and Child Health (CISMAC), University of Bergen, Bergen, Norway; 3 Department of Health Promotion and Development, University of Bergen, Bergen, Norway; 4 Department of Sociology, Addis Ababa University, Addis Ababa, Ethiopia

**Keywords:** Sexual and reproductive health, Abortion law and policy, Contraception, Youth, Sub-Saharan Africa, Ethiopia, Tanzania, Zambia

## Abstract

This editorial provides an overview of a thematic series that brings attention to the persistently deficient and unequal access to sexual and reproductive health services for young women in sub-Saharan Africa. It represents an effort to analyze the multifaceted relationship between laws, policies and access to services in Ethiopia, Zambia and Tanzania. Using a comparative perspective and qualitative research methodology, the papers presented in this issue explore legal, political and social factors and circumstances that condition access to sexual and reproductive health services within and across the three countries. Through these examples we show the often inconsistent and even paradoxical relationship between the formal law and practices on the ground. Particular emphasis is placed on safe abortion services as an intensely politicized issue in global sexual and reproductive health. In addition to the presentation of the individual papers, this editorial comments on the global politics of abortion which represents a critical context for the regional and local developments in sexual and reproductive health policy and care provision in general, and for the contentious issue of abortion in particular.

## Background

### The global politicization of reproductive health

Reproductive health and abortion are highly politicized issues both on global and national levels, and are subject to continuous contestations arising from questions about gender and equity, human rights, morality, religion, and cultural norms. Issues related to reproduction mobilize strong sentiments among social and political groups and carry great symbolic value for governments. All societies exert control over reproduction, but how and with what justification varies. Abortion is a particular case in point. While some countries move their policies and laws in a more liberal direction, others move towards tougher restrictions. Struggles to promote legal and safe abortions globally have met strong resistance from conservative religious movements and action groups, and the absence of abortion in the reproductive health strategies both in the Millennium Development Goals (MDGs) and the Sustainable Development Goals (SDGs) speaks to the low priority placed on safe abortion within the otherwise highly prioritized maternal health agenda.

Transnational networks of actors who fight for more ‘conservative’ sexual and reproductive health policies, and abortion policies in particular, are increasingly active across the globe. We have seen intensive mobilization against abortion rights in the form of concerted cross-country campaigns organized by groups such as Agenda Europe, a conservative religious union of more than a hundred organizations from thirty European countries [1, 2]. The politicized nature of the abortion issue was compellingly illustrated by the reinstatement of the ‘Mexico City Policy’ (commonly referred to as the ‘global gag rule’) by the US President Donald Trump in 2017, a move that severely restricted the provision of development aid to organizations that offer abortion services or provide information about abortion [3, 4]. With USAID as the greatest contributor to reproductive health programmes in the global south, the Mexico City Policy has a great impact on existing SRHR initiatives. A recent article in *The Lancet* by Brooks et al. (2019) [5] documents an increase in illicit abortions in the wake of Trump’s reinstatement of the Mexico City Policy. The authors argue that this increase in clandestine abortions is a consequence of a decreasing use of contraceptives, as the organisations affected by the policy are also important distributors of modern contraceptives. In a commentary in *The Lancet*, two of the authors of the present collection commend Brooks and colleagues for adding much needed documentation of the wide-ranging effects of the Mexico City Policy [6]. However, the authors also remind us of the need to situate global mechanisms - like the Mexico Policy - within the specific contexts where they take effect. They argue that only by considering the complex web of socially, morally, and politically embedded factors, that along with the Mexico City Policy have implications for contraceptive use and abortions, can we gain insight into the mechanisms that ultimately facilitate or block actual access to reproductive health services (ibid). The present thematic issue, which is part of a cross journal collection, sets out to investigate the complexity of intersecting factors that impact on actual access scenarios in three different country contexts in sub-Saharan Africa.

The papers presented spring out of a comparative and transnational research endeavor on competing normative processes and discourses on abortion and fertility control. The project investigated how international initiatives and national policies articulate with local moralities and practices related to fertility control and abortion among adolescents in the respective country contexts of Ethiopia, Zambia and Tanzania. Particular attention was paid to the relationship between national abortion laws and policies, and women’s actual access to safe abortion services in the three countries. With abortion laws differently located on the permissive-restrictive spectrum, our assumption was that a comparative project would yield policy relevant insights with transferable value to other contexts characterized by low adolescent contraceptive use and continued high rates of unwanted pregnancy and unsafe abortion. The papers demonstrate how the dynamics between national abortion laws and policies, and the religious and cultural landscapes in which abortion issues are set, generate unpredictable and at times paradoxical outcomes in terms of actual access to abortion services.

### The unpredictable articulation between national law and access to services

The concept of *reproductive governance* suggested by Morgan and Robert (2012) [7], and the *policy analysis* framework developed by Walt and Gilson [8, 9], have assisted us in moving our analysis of abortion and fertility control beyond legal frameworks to the multiplicity of social and political mechanisms and processes involved in transforming reproductive health policies to ‘on the ground’ practice. The comparative cross-country paper by Blystad and colleagues *The access paradox: the abortion law, policy and practice in Ethiopia, Tanzanian and Zambia* [10] discusses the cultural, social and political conditions that underlie the apparent paradoxical relationship between the national abortion laws, abortion policy and women’s actual access to safe abortion services. While the abortion law in Zambia has been classified as ‘liberal’, access to safe abortion services is severely restricted by a number of formal and informal mechanisms operating on community and health systems levels. By contrast, the highly restrictive law on abortion in Tanzania is negotiated in ways that seems to facilitate access to medical abortion procedures off label. The Ethiopian case exemplifies a law that categorizes abortion as illegal under the criminal law, but which at the same time, is accommodative of safe abortion services which are being rolled out with strong political commitment.

Morgan and Roberts [7] argue that sexuality and reproduction are governed by elusive mechanisms organized in ‘moral regimes’ that cut across multiple scales from personal and intimate behaviours to more public and political judgements. They emphasize how a variety of actors, including state institutions, religious organizations and NGOs use economic and moral mechanisms, power and coercion “*to produce, monitor and control reproductive behaviours and practices”* (7:243). Our three country case studies, provide a deeper analysis of the national discourses surrounding abortion and illustrate how such subtle mechanisms are employed by different actors and institutions fighting to defend their position on abortion.

In their case study from Zambia *Shaping the abortion policy - Competing discourses on the Zambian Termination of Pregnancy Act* [11], Haaland and colleagues challenge the prevailing notion that the Zambian abortion law is liberal. Based on archival and ethnographic material the paper explores the relationship between a legal framework, the moral and political disputes surrounding abortion in this self-proclaimed Christian nation, and access to sexual and reproductive health services. The authors demonstrate how the inherent ambiguity of the law is actively exploited by both the ones who work to limit access to safe and legal abortion services, and by those who work to increase access to safe and legal services.

The various actors’ positions on the abortion law are shaped in policy environments increasingly influenced by global actors and international networks in a dynamic interplay with local norms and values. Sambaiga and colleagues’ case study from Tanzania *Health, Life and Rights: A Discourse Analysis of a Hybrid Abortion Regime in Tanzania* [12] explores the multiplicity of discourses surrounding the abortion issue within the context of a highly restrictive abortion law. The paper problematizes the common notion of the Tanzanian abortion landscape as unambiguously conservative / restrictive. It demonstrates how a hybrid discursive regime on abortion is encountered in today’s Tanzania, and argues that such a discursive regime which is cutting across the restrictive-liberal divide, generates loopholes that ease access to safer abortion services despite a highly restrictive abortion law.

In a changing environment policy making processes also change. While the government remains key in policy making, policy analysis must, as Walt and Gilson [8, 9] have pointed out, also recognize the importance of the context, the multiplicity of actors affecting the process and the unpredictability of the process itself. Tadele and colleagues‘ case study from Ethiopia *An uneasy compromise’: Strategies and dilemmas in realizing a permissive abortion law in Ethiopia* [13] scrutinizes the policy shift that in 2005 relaxed a highly restrictive abortion law in a context of strong anti-abortion public opinion. The paper shows how actors implementing the more permissive abortion policy in a very conservative environment, actively chose a public health approach and a strategy of silence not to provoke anti-abortion sentiments and politicization of the abortion issue.

In the two last papers of this issue, we move from the policy and organisational level to challenges faced by people on the ground grappling with issues of sexuality, reproductive health and abortion in everyday life. With Ethiopia’s relatively recent and permissive abortion law as a backdrop, Zenebe and Haukanes explore how socio-cultural and religious norms surrounding female premarital and gendered and rural-urban inequities, play into the manner in which students handle unintended pregnancies. The article *When abortion is not within reach: Ethiopian university students struggling with unintended pregnancies* [14] reveals how a morally charged landscape produces scenarios of denial located within a web of economic and emotional challenges for female students who become pregnant. The article demonstrates how a shame-silence nexus forcefully operates in the lives of female students who carry a pregnancy to term.

A powerful space of politics is the school. In their paper *‘Why do they want us to teach sexuality education’? Teacher discretion in teaching comprehensive sexuality education in Zambia* [15], Zulu and colleagues investigate teachers’ discretion in implementing an ambitious nation-wide program for comprehensive sexuality education (CSE) based in the ideology of sexual and reproductive health and rights. The curriculum is developed by the Ministry of Education supported by UNESCO. Drawing upon Lipsky’s concept of ‘street level bureaucracy’ (1980) the paper demonstrates how teachers, as street level bureaucrats, not only implement policy, but actively shape policy through their use of discretion in their encounter with pupils (see eg. Bierschenk and de Sardan 2014 [16]; Melberg 2018 [17]). The paper shows a high level of resistance to the curriculum by both teachers and the communities surrounding the schools. Using Lipsky’s insights into the dynamics of discretion, it shows how teachers make their own decisions about how, what and when to teach CSE which in practice implies teaching sexual abstinence.

## Conclusions

The papers in this thematic issue detail how reproductive governance is played out with reference to adolescent sexuality and pregnancy termination in the three country contexts where sexual relations, pregnancy and childbearing out of wedlock are morally condemned in public and religious discourse. A theme that runs through the extensive material is the silence and avoidance that - beyond a few moments of heated political outbursts - commonly surround these topics in public discourse. Even though public silence does facilitate roll out of abortion services to some extent, as the Ethiopian case exemplifies, silence simultaneously slow down or obstruct policy processes. With continued public silence, unsafe abortions are allowed to continue both in restrictive legal contexts and in contexts where more permissive abortion laws are in place. Such dynamics are detrimental for young girls and women and make it even more problematic to deal with unwanted pregnancy in sub-Saharan Africa.

## Data Availability

Not applicable.
